# Quack Extortions—Toothache

**Published:** 1850-01

**Authors:** 


From the London Forceps.

### QUACK EXTORTIONS—TOOTHACHE.

"On Monday week, being troubled with a serious tooth-ache, from which I had suffered for two or three days before," says a correspondent of the *Liverpool Mercury*, "I made up my mind to have it extracted, and, accordingly, directed my steps to a person residing not a hundred miles from Bold-street, who advertises that he has discovered an infallible remedy for the tooth-ache without drawing, and that 'he applies it himself, at a moderate charge, to suit all classes.' When I arrived at the place, the practitioner, with many compliments and ceremonies, informed me that it was bad policy to extract a tooth, and that he would apply a remedy which should prove effectual for twenty years. He then began operating upon my mouth with many instruments. After he had been engaged in this manner for about fifteen minutes, he said he had made a cure—but a cure that was, to me, worse than the disease, for the pain had rather increased than abated. When I asked him the sum I had to pay, he put together as many items as amounted to twenty-six shillings! and was going on, when I stopped him in a fright, assuring him that I was not prepared for any such charge, and that I never expected to pay more than three or four shillings, which I considered would be paying him liberally for the time and trouble he had expended in the operation. All my eloquence was lost upon my doctor, who seemed, like Shylock, determined to have his bond. I, however, could not give him the whole of his exorbitant fee, for I had it not; he, however, took my all, amounting to fifteen shillings, and in less than twenty minutes thereafter my fifteen shillings dropped out of my mouth in the shape of a little cotton wool, with which he had stopped up my teeth. The pain which the fright of his most abominable charge had for a moment driven away, now returned with tenfold vengeance, and was never allayed till I went, in two days, and had the tooth extracted by Mr. Lloyd, of Bold-street, from which operation I received immediate relief, for a sum more commensurate with my means."

The above, from the London Forceps, suits so well the meridian of Cincinnati, that we have thought it worth publishing in the Register.

A few weeks since, a gentleman called at an office of one very much like the dentist living not a hundred miles from Bold-street, (indeed we are not certain but it may be the same individual, for he is from the other side of the "big waters,") and applied for relief of toothache. Some application was made to destroy the nerve, yet the pain continued, and so did the patient, until in the course of two or three days he had paid the dentist some two dollars and a half, when in despair of relief, he determined to have his tooth extracted. But after four or five ineffectual efforts, he gave up this also. In this state, the patient called at our office and desired relief in some way or other, which was speedily afforded with a pair of forceps; and what to us was a matter of some astonishment, was the ease with which the tooth was removed, how it is, that a man professing to be a dentist, should make four or five efforts to remove such a tooth, certainly needs some explanation. We have heard it intimated that this gentleman has a *new and remarkable plan* for inserting teeth. They appear to be fastened by a magnet, which is so powerful that they never come out.

J. T. Cin. Ed.

